# The use of chiropractors by older adults in the United States

**DOI:** 10.1186/1746-1340-15-12

**Published:** 2007-09-06

**Authors:** Fredric D Wolinsky, Li Liu, Thomas R Miller, John F Geweke, Elizabeth A Cook, Barry R Greene, Kara B Wright, Elizabeth A Chrischilles, Claire E Pavlik, Hyonggin An, Robert L Ohsfeldt, Kelly K Richardson, Gary E Rosenthal, Robert B Wallace

**Affiliations:** 1 College of Public Health, The University of Iowa, Iowa City, Iowa, USA; 2 College of Medicine, The University of Iowa, Iowa City, Iowa, USA; 3 Center for Research in the Implementation of Innovative Strategies in Practice (CRIISP), Iowa City Veterans Affairs Medical Center, Iowa City, Iowa, USA; 4 College of Business, The University of Iowa, Iowa City, Iowa, USA; 5 College of Liberal Arts and Sciences, The University of Iowa, Iowa City, Iowa, USA; 6 College of Rural Public Health, Texas A&M University Health Science Center, College Station, Texas, USA

## Abstract

**Background:**

In a nationally representative sample of United States Medicare beneficiaries, we examined the extent of chiropractic use, factors associated with seeing a chiropractor, and predictors of the volume of chiropractic use among those having seen one.

**Methods:**

We performed secondary analyses of baseline interview data on 4,310 self-respondents who were 70 years old or older when they first participated in the Survey on Assets and Health Dynamics Among the Oldest Old (AHEAD). The interview data were then linked to their Medicare claims. Multiple logistic and negative binomial regressions were used.

**Results:**

The average annual rate of chiropractic use was 4.6%. During the four-year period (two years before and two years after each respondent's baseline interview), 10.3% had one or more visits to a chiropractor. African Americans and Hispanics, as well as those with multiple depressive symptoms and those who lived in counties with lower than average supplies of chiropractors were much less likely to use them. The use of chiropractors was much more likely among those who drank alcohol, had arthritis, reported pain, and were able to drive. Chiropractic services did not substitute for physician visits. Among those who had seen a chiropractor, the volume of chiropractic visits was lower for those who lived alone, had lower incomes, and poorer cognitive abilities, while it was greater for the overweight and those with lower body limitations.

**Conclusion:**

Chiropractic use among older adults is less prevalent than has been consistently reported for the United States as a whole, and is most common among Whites, those reporting pain, and those with geographic, financial, and transportation access.

## Background

### Complementary and alternative medicine in the United States

Complementary and alternative medicine (CAM) therapies have existed since antiquity. Serious interest in investigating and evaluating them, however, is a rather recent phenomenon in the United States [1]. Indeed, the 2005 Institute of Medicine (IOM) report on CAM had three principal goals: (1) to describe the use of CAM therapies; (2) to identify issues related to the translation of validated CAM therapies into medical practice; and, (3) to confront the challenges and barriers to conducting rigorous research on the benefits of CAM [2].

The first nationally representative data on CAM use in the United States came from a telephone survey of 1,539 adults conducted in 1990. Those data showed that about one-third of the adults sampled reported some form of CAM use in the past year [3]. Of these, the three most frequent forms of CAM use were relaxation techniques (13%), chiropractic services (10%), and massage therapy (7%) [3]. Five subsequent nationally representative studies of CAM use in the United States [4-8] have reached reasonably comparable prevalence estimates, with one exception. It involved the 2002 National Health Interview Survey (NHIS), from which a 62% prevalence estimate of any CAM use was derived [8]. The NHIS estimate, however, was based principally upon a broader question referring to praying for one's own health, which 43% of that American sample reported doing. This anomaly aside, prevalence estimates for the use of the most identifiable form of CAM – chiropractic services – in the United States have been generally consistent. In fact, the prevalence estimates for chiropractic use from the three largest CAM studies in the United States are very tightly clustered at 6.8%, 7.5%, and 7.6% [4,7,8]. This range is also consistent with the lower end of a more recent descriptive review in this journal of 137 articles that involved chiropractic and CAM utilization [9]. That review found that although rates of chiropractic use varied considerably among these smaller, more parochial studies, they mostly fell within a 6–12% range [9].

As generally consistent as the results of these prior studies of chiropractic use in the United States have been (as well as those of the 137 smaller, more parochial studies recently reviewed in this journal [9]), they are not without limitations. First, each of the six nationally representative studies were based on self-reports. Although chiropractic use may be the most straightforward CAM element for adults to accurately identify and report, we are unaware of any published prevalence estimates derived from nationally representative administrative claims data in the United States. Second, all of the prior studies have focused on the use of chiropractic services in the past year. None have considered longer periods, or whether chiropractic use may be a regular component of an adult's health care. Third, the six prior studies of chiropractic use in the United States do not provide multivariable modeling of factors associated with the use of chiropractors, or the volume of chiropractic visits. Finally, although several of the prior studies include older adults, and most report finding an inverted U-shaped demand curve that peaks among middle aged individuals, none have focused exclusively on nor have rigorously explored chiropractic use among older adults.

### Medicare coverage for chiropractic in the United States

In the United States, private health insurance has historically been employer-based. Indeed, it was only in 1965 when Medicare and Medicaid were introduced as federal programs to extend health care coverage to the poor (especially children) via Medicaid, and via Medicare to those over 65 years of age, because these older adults were likely to have lost their employer-based private health insurance due to retirement. As part of the 1972 Social Security Amendments, Medicare reimbursement was extended to cover chiropractic, but only "for manual manipulation of the spine to correct a subluxation demonstrated by an X-ray," with the further limitation of that coverage to "subluxations that result in a neuromusculoskeletal condition for which manual manipulation is appropriate treatment" [10]. It is important to note here that the evidentiary X-ray requirement is not covered by Medicare in the United States if provided by a chiropractor, and that the X-ray must precede the delivery of chiropractic services. Medicare regulations further limit the scope of what falls under appropriate chiropractic services by explicitly noting that:

" [a] treatment plan that seeks to prevent disease, promote health and prolong and enhance quality of life, or therapy that is performed to maintain or prevent deterioration of a chronic condition is not a Medicare benefit. Once the maximum therapeutic benefit has been achieved for a given condition, ongoing maintenance therapy is not considered to be medically necessary under the Medicare program." [[10]; p 3]

This underscores the importance for chiropractors in the United States to document the initial history, expected treatment duration, treatment frequency, and treatment goals and objectives in the written treatment plan for their Medicare patients. That treatment plan must be maintained as part of the medical record and provided to the Medicare insurance carrier on request. A recent analysis by the United States Office of the Inspector General (OIG) indicates that the principal reason for rejecting chiropractic claims is that they were for *maintenance *treatments, which accounted for about two-thirds of the dollar value of all rejected Medicare chiropractic claims in 2001 [10]. The OIG report also concluded that the volume of chiropractic services was directly related to medical necessity, and identified a threshold of 12 treatments per year as the point beyond which it is increasingly unlikely that individual services could be considered medically necessary. Medicare policy toward chiropractic did not change much for the next 28 years.

The Balanced Budget Act (BBA) of 1997, however, provided a profound change in Medicare chiropractic coverage and reimbursement policy in the United States. Effective January 1, 2000, the BBA removed the pre-existing X-ray requirement, and established guidelines for demonstrating subluxation as "a motion segment, in which alignment, movement integrity, and/or physical function of the spine are altered although contact between joint surfaces remains intact" [10]. The physical exam must identify (a) asymmetry/misalignment or abnormality in the range of motion, *and *(b) either pain/tenderness or associated soft tissue changes. The effect of the BBA has been dramatic [10]. Prior to 2000 the annual percentage of Medicare beneficiaries in the United States who saw a chiropractor was steady at about 4.5%. By 2002, however, the annual prevalence had already risen to 6.0%. The growth in Medicare expenditures for chiropractic services in the United States resulting from the BBA policy change is even more marked, rising from $360 M (USD) in 1999 to $683 M (USD) in 2004, with the number of approved services rising from about 13 M to 21 M [10].

### The purpose of this study

In this article we use administrative claims for calendar years 1991–1996 from the Center for Medicare and Medicaid Services (CMS) in the United States to examine the use of chiropractic among older adults. These CMS claims data were then linked to baseline interviews with a large nationally representative sample of older adults in the United States in order to (a) determine the extent of chiropractic use over a four-year period, (b) identify factors associated with seeing a chiropractor, and (c) evaluate correlates of the volume of chiropractic use among those having seen one.

## Methods

### Data

We conducted a secondary analysis of the baseline interview data from the Survey on Assets and Health Dynamics Among the Oldest Old (AHEAD), which is sponsored by the National Institute on Aging, of the National Institutes of Health (NIH), in the United States. The design and sampling approach in the AHEAD have been well described elsewhere [11-14]. Because African Americans, Hispanics, and Floridians were oversampled, all analyses are weighted to adjust for the unequal probabilities of selection due to the multi-stage cluster- and over-sampling. The AHEAD provides a nationally representative probability sample of the United States that includes 4,310 men and women who were 70 years old or older, were self-respondents at baseline (1993), and whose survey data could be linked to their CMS Medicare claims. CMS Medicare claims were available from January 1991 through December 1996. For each AHEAD subject, we used all CMS Medicare claims available within a four-year window centered on the date of their individual baseline in-home interviews (i.e., two years prior to and two years afterwards).

### Measuring chiropractic use

To identify visits to chiropractors in the CMS Medicare claims, we examined two sources of information. The first involved *Current Procedural Terminology *(CPT) codes for the United States [15]. For the entire period under study, the CPT code that was supposed to be used for all subluxation procedures performed by chiropractors was A2000 ("manual manipulation of the spine to correct a subluxation"). For the 4,310 AHEAD subjects, we found 13,340 line entries containing the A2000 CPT code over the four-year period. Our second source of information was the specialty type code associated with the Unique Physician Identifier Number (UPIN) in the United States. We found that 18,016 line entries contained UPIN specialty codes for chiropractors. When cross-classified, only 284 (2%) of the A2000 CPT code entries were not associated with a chiropractor's UPIN. The majority of these (86%) were associated with ambulatory surgical centers (specialty code 49). Because our focus is on the use of chiropractors, we relied solely on the UPIN specialty codes for chiropractors at the line level in the CMS Medicare claims. We then aggregated up from the line level, defining any visit that included a chiropractic line charge as a visit to a chiropractor. This approach is consistent with CMS Medicare policy in the United States (Title 42, Part 410; 51 FR 41339, 64 FR 59439, and 66 FR 55328).

### Covariates

To model the use of chiropractic among older adults, we chose variables traditionally used in studying the demand for health care [16], namely sociodemographics, socioeconomics, lifestyle, disease history, functional health status, prior health services use, and the supply of providers in the county. All of these data were obtained from the baseline interviews, except for the supply of chiropractors in the county, which was taken from archival sources. Sociodemographic characteristics included age, sex, race, and living arrangements. Given the potential for nonlinear age effects, we used a set of four dummy variables, contrasting those aged 75–79 years old, 80–84 years old, and 85 years old or older with those aged 70–74 years old (the reference group). Sex was a simple contrast of men (coded 1) vs. women (coded 0). Race was measured with a set of three dummy variables contrasting African Americans and Hispanics with Whites (the reference group). Living arrangements were reflected by a marker coded 1 for living alone vs. 0 for living with others.

Socioeconomic status was measured by education, income, veteran status, and having private insurance. Given the age of our subjects, education was coded as a set of dummy variables contrasting only having attended grade school or having some college, with having a high school education (the reference group). Household income was measured with a set of five dummy variables reflecting income quintiles, with the middle one as the reference group. Veteran status was coded 1 for veterans and 0 for nonveterans. We included it because veterans in the United States have access to the Veterans Health Administration (VHA) in addition to Medicare. Having private health insurance, in addition to Medicare coverage, was coded 1 for yes and 0 for no. We included it because those with access to private health insurance might have their chiropractic visits paid for this way rather than from Medicare.

Lifestyle was measured by cigarette smoking, alcohol consumption, their interaction, body mass, and ever having had a valid motor vehicle (driver's) license. Both cigarette smoking and alcohol consumption may be considered coping mechanisms, and thus are quite relevant to the use of chiropractic, which is commonly used in response to pain. Each of these substance use measures were coded 1 if the subject had ever smoked cigarettes or drank alcohol, and 0 if not. We also included the interaction between these two measures (smoking and drinking) to determine whether there was a synergistic effect of such substance use on the demand for chiropractic. Body mass was measured using a set of dummy variables contrasting being overweight or obese with being of normal or underweight status (the pooled reference group) based on established body mass index (BMI) cut-offs. Driving status was a binary variable contrasting those who had never had a valid driver's license (coded 1) with those who at one time had had one (coded 0), because many members of this cohort in the United States never did.

Disease history was obtained by asking each respondent whether they had ever been told by a physician that they had arthritis (affirmative responses mostly reflected osteoarthritis), cancer (excluding minor skin cancer), diabetes, hypertension, lung disease (affirmative responses mostly reflected chronic obstructive pulmonary disease), a heart condition (affirmative responses mostly reflected congestive heart failure or a myocardial infarction), a hip fracture, or a psychological condition (including emotional, nervous, or psychiatric problems). Subjects were also asked if they were often bothered by pain. Each of these was reflected in a binary marker coded 1 for yes and 0 for no. In addition, we included a set of dummy variables to capture the extent of comorbidity, by contrasting having none or two or more of the above diseases vs. having only one (the reference category).

Functional limitations were measured in numerous ways. The first three were simple counts (0–5) of whether the subject reported having any difficulty in performing activities of daily living (ADLs) such as bathing or dressing, performing instrumental ADLs (IADLs) such as money management or taking their medications, or lower body limitations such as stooping, kneeling, or crouching. The next four measures of functional limitations involved binary markers for whether the subject reported fair or poor (as opposed to excellent, very good, or good) responses to questions assessing their hearing, vision, and memory acuity, as well as their overall health. A binary marker was used to reflect whether the subject currently drove a motor vehicle. We also used two multiple item scales to tap depressive symptoms and cognitive function. For depressive symptoms, we used the sum of eight common depressive symptoms taken from the well-established Centers for Epidemiologic Studies Depression (CES-D) scale [17]. These sums were then recoded into a set of dummy variables contrasting having no or three or more symptoms with having 1–2 symptoms (the reference group). For cognitive status, we used the well-established Telephone Interview for Cognitive Status (TICS-7) battery [18]. The TICS-7 score was than recoded into a set of dummy variables contrasting 0–10 (low performance) and 14–15 (high performance) with normal performance (11–13) as the reference group.

The two final categories of covariates were the use of health services, and the supply of chiropractors in the community. There were two measures of self-reported health services use – the number of physician visits in the year prior to baseline, and whether or not the subject had continuity of care. The latter was defined as having no more than 8 months between visits to the same physician during the two years prior to the baseline interview [13]. The supply of chiropractors was taken from a well-established archival data source for area (geo-political) markers in the United States, known as the *Area Resource File*. The supply of chiropractors per thousand persons in the county was coded into tertiles, with the middle tertile used as the reference group.

### Analytic approach

We used multivariable logistic regression to model whether these CMS Medicare beneficiaries had one or more visits to a chiropractor over the four-year period (two years before their baseline interview and two years after their baseline interview), and followed standard procedures for model development and evaluation [19-22]. To examine factors associated with the volume of chiropractic visits among those having one or more, we used multivariable negative binomial regression [23,24]. Both sets of multivariable analyses included the sociodemographic, socioeconomic, lifestyle, disease history, functional health status, prior health services use, and the supply of providers in the county variables.

## Results

### Descriptive

Among the 4,310 AHEAD subjects in the analytic sample (weighted N = 4,337), the mean age was 77 years old, 35% were men, 9% were African American, 4% were Hispanic, and 43% were widowed. Mean income was $25 K (USD), and one-fourth had only been to grade school. One-fourth reported arthritis, 8% reported angina, 13% reported cancer, 11% reported diabetes, 46% reported hypertension, 4% reported fractured a hip, and 7% reported psychological problems. The mean number of ADLs was 0.29 and the mean number of IADLs was 0.18. Further data about this analytic sample are available elsewhere [13,14,25].

### Chiropractic use

The mean annual percentage of subjects in this United States sample having any chiropractic visits was 4.6% (range = 4.0% to 5.1%), with no evidence of any secular trend. To provide criterion validation for our classification approach [26], Table 1 contains the percentage distribution of the six most frequent primary ICD9-CM (*International Classification of Diseases, 9^th ^Revision, Clinical Modification*) codes for CPT code A2000. We used ICD9-CM codes (rather than the newer ICD10-CM codes), because ICD9-CM codes were used in CMS Medicare claims for the 1991–1996 period under study. As shown, the top six ICD9-CM codes accounted for over 92% of all chiropractic visits in each year, and there was no evidence of any secular trend. Moreover, all of the ICD9-CM codes are within the legitimate domain of chiropractic.

**Table 1:** Percentage Distribution of the Six Most Frequent Primary ICD9-CM Codes for CPT Code A2000 (Manual Manipulation of the Spine to Correct a Subluxation), by Calendar Year.

ICD9-CM Code	Description	1992	1993	1994	1995	1996
839	Other, multiple, and ill-defined dislocations	33.9	39.2	41.5	45.5	38.1
739	Nonallopathic lesions, not elsewhere classified	21.3	26.1	20.0	19.6	34.7
724	Other and unspecified disorders of back	16.0	13.1	15.3	**10.4**	11.9
723	Other disorders of cervical region	10.8	6.6	5.9	12.1	**2.2**^b^
847	Sprains and strains of other and unspecified parts of back	5.6	4.3	**3.1**	5.3	3.3
722	Intervertebral disc disorders	4.7	**3.4**^a^	5.6	1.5	3.1
	Cumulative Percentage	92.2	92.7	91.3	94.3	93.2

^a^Code 729 was slightly more frequent (3.5%).

^b^Code 729 was slightly more frequent (3.1%), as was code 721 (2.3%).

Cells with **bold-faced **entries indicate divergence from the 1992 frequency pattern.

The four-year (two years before and two years after each baseline interview) period prevalence rate in this United States sample of subjects having any chiropractic visits was 10.3%. Among those with one or more visits to a chiropractor during the four-year period, the mean number of visits for that four-year period was 17.9 (SD = 28.6). About half (48%) of the subjects who had seen a chiropractor saw her or him during only one particular calendar year. However, 21.7% saw a chiropractor during two calendar years, 10.6% saw a chiropractor during three calendar years, and 19.7% saw a chiropractor during four or more calendar years.

### Multiple logistic regression models

Table 2 [see Additional File 1] contains the adjusted odds ratios (AORs) obtained from modeling whether or not a chiropractor was seen during the four-year period (two years before and after each subject's baseline interview). The first model (Model 1) included only the sociodemographic characteristics, with subsequent models sequentially adding the socioeconomic, lifestyle, disease history, functional health status, prior health services use, and the chiropractic supply variables. Also shown are the results from a stepwise model (Model 7). Although our focus is on the results shown in Model 6, which includes all of the independent variables, the consistency of the AORs shown in Models 1–5 and 7 demonstrate that neither meaningful effect decomposition nor harmful multicollinearity were present [21].

Among the sociodemographic characteristics, Model 6 indicates that African Americans (AOR = 0.239, p < .001) and Hispanics (AOR = 0.454, p < .05) were substantially less likely than their White counterparts to have seen a chiropractor. None of the socioeconomic characteristics had statistically significant effects, although there was a trend (p < .10) for those with lower educational attainment and private insurance to have been more likely to have seen a chiropractor, while veterans were less likely to have done so. The only significant association among the lifestyle factors was that those who drank alcohol were more likely to have visited a chiropractor. Among the disease markers, subjects who reported being bothered by pain were substantially more likely to have seen a chiropractor (AOR = 1.752, p < .001), and there was a trend (p < .10) for a greater likelihood of chiropractic use among those with arthritis. Two of the functional status measures were substantially associated with seeing a chiropractor – the ability to drive a car substantially increased the likelihood (AOR = 1.767, p < .01), while having 3 or more depressive symptoms decreased it (AOR = 0.694, p < .05). There was also a trend (p < .10) for those with more IADL limitations to be less likely to have seen a chiropractor. Finally, the use of chiropractors was less likely in counties where their supply was in the lower tertile (AOR = 0.700, p < .01). Overall, Model 6 fit the data reasonably well, with a C-statistic of .688, which was only reduced to .675 in the stepwise-trimmed analysis (Model 7).

### Negative binomial regression models

Table 3 [see Additional File 2] contains the adjusted means ratios (AMRs) obtained from modeling the number of chiropractic visits among the 446 AHEAD subjects who had one or more during the four-year period. As with Table 2, six sequential models are shown, along with the results of a stepwise model. And once again, although our focus is on the results shown in Model 6 (which includes all of the independent variables), the consistency of the AMRs shown in all the models demonstrates that effect decomposition and multicollinearity were not a problem [23,24].

Among the sociodemographic variables, only those who lived alone had substantially fewer visits to chiropractors (AMR = 0.737, p < .05), although, there was a trend (p < .10) for the oldest old and Hispanics to have fewer chiropractic visits as well. Income was the only socioeconomic characteristic associated with the volume of chiropractic visits – those in the two lowest income quintiles had substantially fewer visits (AMRs = 0.519 and 0.689, p < .01 and <.05, respectively) than their more affluent counterparts. Among the lifestyle factors, those who were overweight (compared to those who were of normal weight or underweight, the pooled reference group) had significantly more visits to the chiropractor (AMR = 1.262, p < .05). The only disease marker that was associated with the number of chiropractic visits was having a history of hip fracture, with those who had a hip fracture prior to baseline having only about half as many chiropractic visits as their counterparts (AMR = 0.535, p < .05). Among the functional status measures, those with lower body limitations had substantially higher chiropractic use rates (AMR = 1.171 per limitation, p < .01). Finally, subjects with poor cognitive status had substantially fewer chiropractic visits (AMR = 0.687, p < .05). The pseudo R-squared value of .214 indicates that the final model was robust [23,24].

## Discussion

In this article, we determined the extent of chiropractic use over a four-year period in a large, nationally representative sample of CMS Medicare beneficiaries in the United States. We also identified factors associated with seeing a chiropractor, and evaluated correlates of the volume of chiropractic use among those having seen one. Based on administrative claims data, we found a mean annual prevalence rate of having one or more chiropractic visits of 4.6%, and a four-year period prevalence rate of 10.3%, with no evidence of secular trend. Both of our prevalence estimates are remarkably comparable to the annual rates reported in the three largest prior studies in the United States (6.8%, 7.5%, and 7.6% [4,7,8]). Moreover, our annual prevalence rate is nearly identical to that reported by the United States OIG for all Medicare beneficiaries from the early 1990s through 1999, after which the pre-existing X-ray requirement was removed and annual chiropractic use rates rose to 6% in just two years [27]. The modest difference between our (and the OIG) annual prevalence rates and the self-reported rates from these three larger surveys likely results from the use of chiropractic services that did not result in Medicare claims, either because of private insurance coverage or out-of-pocket payments.

Our study is also the first in the United States to address whether or not chiropractic use is a regular component of an adult's health care. Among those with one or more visits to a chiropractor during the four-year period, about half (48%) only saw a chiropractor during one single calendar year. But, we found that 30.3% of those with chiropractic visits had used a chiropractor in at least three different calendar years. Because we did not find any association between physician visits in the year prior to baseline on the one hand, and either going to see a chiropractor or the number of chiropractic visits on the other hand, chiropractic use in the United States may well be a regular component of an adult's health care that appears not to substitute for the overall volume of physician services [28-30].

The findings from our multivariable models were also informative. African Americans and Hispanics, as well as those with multiple depressive symptoms and those who lived in counties with lower than average supplies of chiropractors were much less likely to use them. The use of chiropractors was much more likely among those who drank alcohol, had arthritis, reported pain, and were able to drive. Among those who saw chiropractors, the volume of visits was lower for those who lived alone, had lower incomes, and poorer cognitive abilities, while it was greater for the overweight and those with lower body limitations. These findings are generally consistent with previous reports that have identified those in pain, younger individuals, Whites, and those with better access (socioeconomic status, insurance coverage, and residing in areas with greater chiropractor to population ratios) as more likely to have used chiropractors [31-41]. We did not, however, find support for prior reports that those who used chiropractors had fewer chronic conditions and less continuity of care (i.e., a regular source of health care) than their counterparts [37-41].

## Conclusion

With one major exception, our results suggest that the use of chiropractic services in the United States is generally rational – that is, people who go to see chiropractors are much more likely to be in pain, and to have geographic, transportation, and financial access to them. Moreover, when seen, chiropractors are used for procedures that are clearly appropriate to their clinical expertise. The major exception to this rational pattern involves the racial disparities in chiropractic use – African Americans and Hispanics are simply much less likely to visit chiropractors than Whites in the United States. Although this relationship has been frequently reported in the literature, it remains unexplained.

## Limitations

Although insightful, our study of chiropractic use by older adults in the United States has several limitations. First, we relied on only four years of claims data from the early 1990s, and thus we were unable to examine changes in demand associated with the implementation of the chiropractic rule changes contained in the BBA in 2000. Second, we focused simply on whether or not any chiropractic services had been used during that period, as well as the number of chiropractic visits among those with at least one. Third, we did not develop a classification system characterizing chiropractic use over time, nor did we explore in sufficient detail whether chiropractic use was a regular component of health care, and if so, for what subset of older adults. Fourth, our approach also failed to consider the potential for selection bias by focusing on the subset of AHEAD subjects with linked Medicare claims, or attrition from Medicare claims due to a subject's movement into managed care, or death. Finally, we barely scratched the surface of whether meaningful comorbidity differentials exist, or the substitutive vs. adjuvant nature of chiropractic services relative to physician services, and we did not address at all the effect of chiropractic use on subsequent health status and health services utilization. Thus, although promising, our results are not definitive.

Therefore, further research is necessary. In particular, we have requested funding from the National Center for Complementary and Alternative Medicine (NCAM), which is a component of the United States NIH, to develop a meaningful and clinically relevant classification system that characterizes chiropractic use patterns over time, their antecedents, and their consequences, as well as to adequately explore several specific issues in the literature [42]. Furthermore, we believe that the research agenda we have proposed to NCAM is entirely consistent with the concluding recommendation of the IOM that, in order to evaluate the value of CAM and chiropractic, there is a compelling need for studies which use "innovative methods of evaluation...for the generation and interpretation of evidence [[2]; p.278]."

## Competing interests

The author(s) declare that they have no competing interests.

## Authors' contributions

FDW conceived of the study, wrote all three grant applications, designed the analyses, interpreted the results, and drafted and revised the manuscript. LL and TRM cleaned and linked all of the data files, and conducted all of the statistical analyses at FDW's direction. KKR and CEP harvested the geocoded data. HA, EAC, RLO, and JFG assisted in the design and oversight of the statistical analyses and their interpretation. EAC and BRG reviewed Medicare regulations pertinent to chiropractic reimbursement. GER and RBW participated in the conceptualization of the grants applications and the overall study design, provided clinical expertise at all stages of the analysis, and assisted in framing the discussion. All authors read and approved the final manuscript.

## Supplementary Material

Additional file 1 Table 2. This file contains Table 2, the Adjusted Odds Ratios from Multivariable Logistic Regressions Predicting Any Use of a Chiropractor During the Four-Year Period (Weighted N = 4,337 Self-Respondents). Click here for file

Additional file 2 Table 3. This file contains Table 3, the Adjusted Means Ratios from Multivariable Negative Binomial Regressions Predicting the Number of Chiropractic Visits During the Four-Year Period (Weighted N = 446 Self-Respondents). Click here for file
