# Supplementary material for: The use of chiropractors by older adults in the United States
Source: Chiropr Osteopat. 2007 Sep 6;15:12. doi: 10.1186/1746-1340-15-12 (PMC2034378; doi:10.1186/1746-1340-15-12)
Supplement: Additional file 1 — Table 2. This file contains Table 2, the Adjusted Odds Ratios from Multivariable Logistic Regressions Predicting Any Use of a Chiropractor During the Four-Year Period (Weighted N = 4,337 Self-Respondents). [file 1746-1340-15-12-S1.doc]

Table 2. Adjusted Odds Ratios from Multivariable Logistic Regressions Predicting Any Use of

a Chiropractor During the Four-Year Period (Weighted N =4,337 Self-Respondents).

| **Variables** | **Model 1** | **Model 2** | **Model 3** | **Model 4** | **Model 5** | **Model 6** | **Model 7** |
| --- | --- | --- | --- | --- | --- | --- | --- |
| **Sociodemographic** |  |  |  |  |  |  |  |
| Age |  |  |  |  |  |  |  |
| <=74 years old (ref) | 1.000 | 1.000 | 1.000 | 1.000 | 1.000 | 1.000 |  |
| 75-79 years old | 0.938 | 0.936 | 0.950 | 0.943 | 1.010 | 0.996 |  |
| 80-84 years old | 1.067 | 1.082 | 1.098 | 1.080 | 1.229 | 1.203 |  |
| >=85 years old | 0.815 | 0.846 | 0.860 | 0.817 | 1.105 | 1.047 |  |
| Men | 1.087 | 1.247 | 1.252 | 1.341 | 1.236 | 1.221 |  |
| Race |  |  |  |  |  |  |  |
| White (ref) | 1.000 | 1.000 | 1.000 | 1.000 | 1.000 | 1.000 |  |
| African American | 0.170*** | 0.218*** | 0.228*** | 0.223*** | 0.219*** | 0.239*** | 0.246*** |
| Hispanic | 0.351** | 0.465* | 0.496+ | 0.467+ | 0.464+ | 0.454* | 0.501+ |
| Living Alone | 0.843 | 0.949 | 0.930 | 0.943 | 0.875 | 0.870 |  |
| **Socioeconomic** |  |  |  |  |  |  |  |
| Education |  |  |  |  |  |  |  |
| Grade school |  | 1.115 | 1.161 | 1.172 | 1.257 | 1.308+ | 1.301+ |
| High school (ref) |  | 1.000 | 1.000 | 1.000 | 1.000 | 1.000 |  |
| Some college |  | 1.055 | 1.005 | 1.035 | 1.021 | 1.017 |  |
| Income |  |  |  |  |  |  |  |
| Lowest quintile |  | 0.674+ | 0.740 | 0.703 | 0.846 | 0.862 |  |
| Second quintile |  | 0.981 | 0.915 | 0.880 | 0.942 | 0.946 |  |
| Third quintile (ref) |  | 1.000 | 1.000 | 1.000 | 1.000 | 1.000 |  |
| Fourth quintile |  | 1.080 | 1.051 | 1.028 | 1.029 | 1.010 |  |
| Highest quintile |  | 1.196 | 1.142 | 1.115 | 1.091 | 1.088 |  |
| Veteran |  | 0.679* | 0.699* | 0.703* | 0.719 | 0.728 |  |
| Private Insurance |  | 1.481* | 1.408* | 1.444* | 1.352+ | 1.335+ | 1.396* |
| **Lifestyle** |  |  |  |  |  |  |  |
| Smoker (ever) |  |  | 0.803 | 0.800 | 0.813 | 0.812 |  |
| Drinker (ever) |  |  | 1.504** | 1.533** | 1.471** | 1.430* | 1.569*** |
| Smoker * Drinker |  |  | 1.000 | 1.006 | 0.976 | 0.942 |  |
| Body Mass Index (BMI) |  |  |  |  |  |  |  |
| Normal/under weight (ref) | |  | 1.000 | 1.000 | 1.000 | 1.000 |  |
| Over weight |  |  | 1.162 | 1.121 | 1.087 | 1.077 |  |
| Obese |  |  | 0.995 | 0.921 | 0.920 | 0.925 |  |
| Never Driven |  |  | 0.997 | 1.014 | 1.024 | 1.019 |  |
| **Diseases** |  |  |  |  |  |  |  |
| Arthritis |  |  |  | 1.253 | 1.377+ | 1.378+ | 1.270+ |
| Cancer |  |  |  | 0.740 | 0.763 | 0.739 | 0.737+ |
| Diabetes |  |  |  | 0.925 | 1.045 | 1.030 |  |
| Hypertension |  |  |  | 0.969 | 0.993 | 1.002 |  |
| Lung disease |  |  |  | 0.701 | 0.768 | 0.794 |  |
| Heart condition |  |  |  | 0.978 | 1.070 | 1.065 |  |
| Hip fracture |  |  |  | 0.797 | 0.926 | 0.970 |  |
| Psychological cond. |  |  |  | 0.943 | 1.056 | 1.071 |  |
| Pain |  |  |  | 1.595*** | 1.813*** | 1.788*** | 1.752*** |
| # of above diseases |  |  |  |  |  |  |  |
| None |  |  |  | 1.007 | 1.025 | 1.071 |  |
| One (ref) |  |  |  | 1.000 | 1.000 | 1.000 |  |
| Two or more |  |  |  | 1.001 | 1.025 | 1.071 |  |
| **Functional Limitations** |  |  |  |  |  |  |  |
| # ADLs w/difficulty |  |  |  |  | 0.857 | 0.860 |  |
| # IADLs w/difficulty |  |  |  |  | 0.827+ | 0.821+ | 0.809* |
| # Lower Body limits |  |  |  |  | 0.972 | 0.973 |  |
| Hearing – poor or fair |  |  |  |  | 1.038 | 1.051 |  |
| Vision – poor or fair |  |  |  |  | 0.824 | 0.846 |  |
| Memory – poor or fair |  |  |  |  | 0.931 | 0.908 |  |

| **Variables** | **Model 1** | **Model 2** | **Model 3** | **Model 4** | **Model 5** | **Model 6** | **Model 7** |
| --- | --- | --- | --- | --- | --- | --- | --- |
| Health – poor or fair |  |  |  |  | 1.049 | 1.019 |  |
| Able to Drive |  |  |  |  | 1.691** | 1.767** | 1.952*** |
| CESD8 Score |  |  |  |  |  |  |  |
| 0 |  |  |  |  | 0.835 | 0.839 |  |
| 1-2 (ref) |  |  |  |  | 1.000 | 1.000 |  |
| 3+ |  |  |  |  | 0.701* | 0.694* | 0.683** |
| TICS7 Score |  |  |  |  |  |  |  |
| 0-10 |  |  |  |  | 1.146 | 1.161 |  |
| 11-13 (ref) |  |  |  |  | 1.000 | 1.000 |  |
| 14-15 |  |  |  |  | 1.112 | 1.111 |  |
| **Self-Reported Use**  # of physician visits in the year before baseline |  |  |  |  |  | 1.011 |  |
| Continuity of care |  |  |  |  |  | 1.156 |  |
| **Supply of Chiropractors** |  |  |  |  |  |  |  |
| Chiropractors per 1,000 |  |  |  |  |  |  |  |
| Lowest tertile (<0.16) |  |  |  |  |  | 0.700** | 0.701** |
| Middle tertile (ref.) |  |  |  |  |  | 1.000 |  |
| Highest tertile (>0.25) |  |  |  |  |  | 0.913 |  |
| **Hosmer-Lemeshow p** | .061 | .744 | .818 | .095 | .359 | .581 | .022 |
| **C-Statistic** | .596 | .620 | .645 | .664 | .685 | .688 | .675 |
|  |  |  |  |  |  |  |  |

^+^ = p < .10

* = p < .05

** = p < .01

*** = p < .001
