# Supplementary material for: The use of chiropractors by older adults in the United States
Source: Chiropr Osteopat. 2007 Sep 6;15:12. doi: 10.1186/1746-1340-15-12 (PMC2034378; doi:10.1186/1746-1340-15-12)
Supplement: Additional file 2 — Table 3. This file contains Table 3, the Adjusted Means Ratios from Multivariable Negative Binomial Regressions Predicting the Number of Chiropractic Visits During the Four-Year Period (Weighted N = 446 Self-Respondents). [file 1746-1340-15-12-S2.doc]

Table 3. Adjusted Means Ratios from Multivariable Negative Binomial Regressions Predicting the Number of

Chiropractic Visits During the Four-Year Period (Weighted N =446 Self-Respondents).

| **Variables** | **Model 1** | **Model 2** | **Model 3** | **Model 4** | **Model 5** | **Model 6** | **Model 7** |
| --- | --- | --- | --- | --- | --- | --- | --- |
| **Sociodemographic** |  |  |  |  |  |  |  |
| Age |  |  |  |  |  |  |  |
| <=74 years old (ref) | 1.000 | 1.000 | 1.000 | 1.000 | 1.000 | 1.000 |  |
| 75-79 years old | 1.062 | 1.099 | 1.079 | 1.122 | 1.105 | 1.082 |  |
| 80-84 years old | 0.823 | 0.950 | 0.953 | 0.997 | 0.979 | 0.945 |  |
| >=85 years old | 0.865 | 1.021 | 1.068 | 1.044 | 1.111 | 1.110 |  |
| Men | 0.850 | 0.674** | 0.692* | 0.758+ | 0.765+ | 0.745+ |  |
| Race |  |  |  |  |  |  |  |
| White (ref) | 1.000 | 1.000 | 1.000 | 1.000 | 1.000 | 1.000 |  |
| African American | 0.933 | 0.825 | 0.824 | 0.721 | 0.775 | 0.731 |  |
| Hispanic | 0.422* | 0.520 | 0.482+ | 0.433* | 0.495+ | 0.502+ | 0.421* |
| Living Alone | 0.669*** | 0.778+ | 0.808 | 0.814 | 0.744* | 0.737* | 0.790* |
| **Socioeconomic** |  |  |  |  |  |  |  |
| Education |  |  |  |  |  |  |  |
| Grade school |  | 0.928 | 0.945 | 1.017 | 1.080 | 1.074 |  |
| High school (ref) |  | 1.000 | 1.000 | 1.000 | 1.000 | 1.000 |  |
| Some college |  | 1.072 | 1.109 | 1.111 | 1.138 | 1.174 |  |
| Income |  |  |  |  |  |  |  |
| Lowest quintile |  | 0.599* | 0.582* | 0.534** | 0.522** | 0.519** | 0.611* |
| Second quintile |  | 0.769 | 0.715* | 0.682* | 0.695* | 0.687* | 0.758* |
| Third quintile (ref) |  | 1.000 | 1.000 | 1.000 | 1.000 | 1.000 |  |
| Fourth quintile |  | 1.078 | 1.042 | 0.996 | 0.889 | 0.867 |  |
| Highest quintile |  | 1.272 | 1.209 | 1.114 | 1.096 | 1.070 | 1.217+ |
| Veteran |  | 1.272 | 1.283 | 1.284 | 1.335+ | 1.331+ |  |
| Private Insurance |  | 0.836 | 0.877 | 0.883 | 0.813 | 0.795 |  |
| **Lifestyle** |  |  |  |  |  |  |  |
| Smoker (ever) |  |  | 0.908 | 1.017 | 1.018 | 1.051 |  |
| Drinker (ever) |  |  | 0.883 | 0.943 | 0.995 | 1.006 |  |
| Smoker * Drinker |  |  | 0.795 | 0.874 | 0.894 | 0.904 |  |
| Body Mass Index (BMI) |  |  |  |  |  |  |  |
| Normal/under weight (ref) | |  | 1.000 | 1.000 | 1.000 | 1.000 |  |
| Over weight |  |  | 1.276* | 1.283* | 1.261* | 1.262* |  |
| Obese |  |  | 1.397* | 1.317 | 1.173 | 1.167 |  |
| Never Driven |  |  | 0.902 | 0.857 | 0.777 | 0.739 |  |
| **Diseases** |  |  |  |  |  |  |  |
| Arthritis |  |  |  | 1.458* | 1.261 | 1.257 | 1.228+ |
| Cancer |  |  |  | 0.841 | 0.873 | 0.891 |  |
| Diabetes |  |  |  | 0.819 | 0.717+ | 0.712+ |  |
| Hypertension |  |  |  | 1.187 | 1.193 | 1.195 |  |
| Lung disease |  |  |  | 0.780 | 0.672 | 0.658+ | 0.672* |
| Heart condition |  |  |  | 0.935 | 0.884 | 0.905 | 0.743** |
| Hip fracture |  |  |  | 0.535* | 0.532* | 0.535* | 0.486** |
| Psychological cond. |  |  |  | 1.179 | 1.170 | 1.147 |  |
| Pain |  |  |  | 1.101 | 0.943 | 0.957 |  |
| # of above diseases |  |  |  |  |  |  |  |
| None |  |  |  | 1.090 | 1.053 | 1.073 |  |
| One (ref) |  |  |  | 1.000 | 1.000 | 1.000 |  |
| Two or more |  |  |  | 0.905 | 0.927 | 0.913 |  |
| **Functional Limitations** |  |  |  |  |  |  |  |
| # ADLs w/difficulty |  |  |  |  | 1.033 | 1.007 |  |
| # IADLs w/difficulty |  |  |  |  | 0.974 | 0.985 |  |
| # Lower Body limitations |  |  |  |  | 1.181*** | 1.171** | 1.178*** |
| Hearing – poor or fair |  |  |  |  | 0.997 | 0.866 |  |
| Vision – poor or fair |  |  |  |  | 1.170 | 1.138 |  |
| Memory – poor or fair |  |  |  |  |  |  |  |

| **Variables** | **Model 1** | **Model 2** | **Model 3** | **Model 4** | **Model 5** | **Model 6** | **Model 7** |
| --- | --- | --- | --- | --- | --- | --- | --- |
| Health – poor or fair |  |  |  |  | 0.870 | 0.893 |  |
| Able to Drive |  |  |  |  | 1.172 | 1.195 |  |
| CESD8 Score |  |  |  |  |  |  |  |
| 0 |  |  |  |  | 1.142 | 1.143 |  |
| 1-2 (ref) |  |  |  |  | 1.000 | 1.000 |  |
| 3+ |  |  |  |  | 0.995 | 1.016 |  |
| TICS7 Score |  |  |  |  |  |  |  |
| 0-10 |  |  |  |  | 0.699* | 0.687* | 0.774* |
| 11-13 (ref) |  |  |  |  | 1.000 | 1.000 |  |
| 14-15 |  |  |  |  | 0.860 | 0.839 |  |
| **Self-Reported Use**  # of physician visits in the year before baseline |  |  |  |  |  | 1.004 |  |
| Continuity of care |  |  |  |  |  | 1.084 |  |
| **Supply of Chiropractors** |  |  |  |  |  |  |  |
| Chiropractors per 1,000 |  |  |  |  |  |  |  |
| Lowest tertile (<0.16) |  |  |  |  |  | 1.127 |  |
| Middle tertile (ref.) |  |  |  |  |  | 1.000 |  |
| Highest tertile (>0.25) |  |  |  |  |  | 1.069 |  |
| **Pseudo R-Squared** | .053 | .096 | .116 | .159 | .204 | .214 | .157 |
|  |  |  |  |  |  |  |  |

^+^ = p < .10

* = p < .05

** = p < .01

*** = p < .001
